# New Insights Into the Skin Microbial Communities and Skin Aging

**DOI:** 10.3389/fmicb.2020.565549

**Published:** 2020-10-26

**Authors:** Zichao Li, Xiaozhi Bai, Tingwei Peng, Xiaowei Yi, Liang Luo, Jizhong Yang, Jiaqi Liu, Yunchuan Wang, Ting He, Xujie Wang, Huayu Zhu, Hongtao Wang, Ke Tao, Zhao Zheng, Linlin Su, Dahai Hu

**Affiliations:** ^1^Department of Burns and Cutaneous Surgery, Xijing Hospital, Fourth Military Medical University, Xi’an, China; ^2^Department of Cardiology, Tangdu Hospital, Fourth Military Medical University, Xi’an, China; ^3^West China School of Public Health and West China Fourth Hospital, Sichuan University, Chengdu, China; ^4^Department of Plastic Surgery, Xijing Hospital, Fourth Military Medical University, Xi’an, China

**Keywords:** skin microbiomes, intrinsic skin aging, photoaging, VISIA, skin immune regulation

## Abstract

Although it is well-known that human skin aging is accompanied by an alteration in the skin microbiota, we know little about how the composition of these changes during the course of aging and the effects of age-related skin microbes on aging. Using 16S ribosomal DNA and internal transcribed spacer ribosomal DNA sequencing to profile the microbiomes of 160 skin samples from two anatomical sites, the cheek and the abdomen, on 80 individuals of varying ages, we developed age-related microbiota profiles for both intrinsic skin aging and photoaging to provide an improved understanding of the age-dependent variation in skin microbial composition. According to the landscape, the microbial composition in the Children group was significantly different from that in the other age groups. Further correlation analysis with clinical parameters and functional prediction in each group revealed that high enrichment of nine microbial communities (i.e., *Cyanobacteria*, *Staphylococcus*, *Cutibacterium, Lactobacillus*, *Corynebacterium*, *Streptococcus*, *Neisseria*, *Candida*, and *Malassezia*) and 18 pathways (such as *biosynthesis of antibiotics*) potentially affected skin aging, implying that skin microbiomes may perform key functions in skin aging by regulating the immune response, resistance to ultraviolet light, and biosynthesis and metabolism of age-related substances. Our work re-establishes that skin microbiomes play an important regulatory role in the aging process and opens a new approach for targeted microbial therapy for skin aging.

## Introduction

Skin is a complex barrier with a variety of biological functions. It provides an important surface for interactions with the external environment and protects the body from pathogenic, chemical, and physical assaults (Proksch et al., 2008). Skin microbiota (bacteria, fungi, and viruses) are indispensable parts of the skin barrier; they regulate inflammatory processes and provide innate and adaptive immunity (Zhai et al., 2018). Microecologically related research is a new direction in the study of skin aging. A range of cutaneous pathological states occurs when the compositional balance of the skin microbiome is broken due to factors such as aging (Kong et al., 2012; Schommer and Gallo, 2013; Prescott et al., 2017; Tett et al., 2017; Rocha and Bagatin, 2018). In addition, the composition of skin microbiomes varies depending on internal and external factors such as skin integrity and physiological status (Dreno et al., 2016), antibacterial therapy (Langdon et al., 2016), and demographic characteristics (Leung et al., 2016b). Skin aging is a process of alterations in the cutaneous structure and physiological changes (Lei et al., 2017; Bonté et al., 2019), accompanied by increases in species richness and changes in the dominant bacteria, according to some studies (Shibagaki et al., 2017). Therefore, we need to prepare a comprehensive picture of the variation in human skin microbiota associated with skin aging to reveal the patterns of interaction between the microbiota and the aging process.

Many studies (Shibagaki et al., 2017; Wilantho et al., 2017; Juge et al., 2018) have confirmed that the skin bacterial microbiomes differ between young women and older women; however, there are limited studies on the variation in the patterns of skin fungal microbiomes with aging. Additionally, existing studies have focused mainly on site-specific anatomic variation in the microbiomes, instead of differences in the aging process on the basis of exposure factors, i.e., intrinsic aging and photoaging. Intrinsic aging is basically the natural aging process of the skin and is determined by internal factors. Photoaging is accelerated aging of the skin caused by excessive exposure to the sun. In photoaging, the skin microbiome is characterized by ultraviolet (UV)-generated reactive oxygen species (ROS), which regulate gene expression related to collagen degradation and elastin accumulation. These ROS affect DNA or decrease protein tyrosine phosphatases, resulting in the upregulation of matrix metalloproteinase production, contributing to photooxidative skin aging and skin cancer (Wlaschek et al., 2001; Gonzaga, 2009). In short, intrinsic skin aging and photoaging are different processes. There is little evidence that microbiomes influence the aging process of the skin, and little is known about the specific variation patterns of microbiota and their functions in the two different aging processes. In an attempt to remedy this lack, we used 16S ribosomal DNA (rDNA) and internal transcribed spacer (ITS) rDNA sequencing to create profiles for exposed and non-exposed anatomical sites, respectively. We measured differences in the characteristics of microbial composition brought about by aging among four different age groups. Combined with clinical parameters, we focused on the functions of specific age-related microbial communities in each aging stage; these functions may be considered key regulators of skin aging. Combinations of microbial communities and age-related pathways in different age groups could affect UV-induced damage, immune barrier integrity, and the biosynthesis and metabolism of substances that alter the aging process. Our findings could be a milestone in targeted microbial anti-aging skin therapy, which would be a benefit to health care for aging-relevant cutaneous idiopathic diseases and wounds in different age groups.

## Materials and Methods

### Skin Sample Collection

Skin samples were collected from 80 healthy participants belonging to four age groups in Xi’an City (Shaanxi Province, China). Each group was composed of 20 individuals. The make-up of the individual groups, including age range and source of skin samples, was as follows: (1) 20 children, 3–7 years (cheek [CCHG], abdomen [ACHG]); (2) 20, youths, 19–23 years (cheek [CYHG], abdomen [AYHG]); (3) 20 middle-aged individuals, 37–42 years (cheek [CMAG], abdomen [AMAG]); and (4) 20 elderly individuals, 65–74 years (cheek [CELG], abdomen [AELG]). Each group was composed of 10 males and 10 females. The study was compliant with the Declaration of Helsinki Principles, and we obtained informed consent from participants or, in the case of the children, from their parents or other relatives, about the significance of the research and the process of implementation. All the participants were notified that the material they provided could identify their health status as that of having no current cutaneous disorders. Persons were excluded if they were receiving antimicrobial therapy or cleaning within 1 month. Participants were asked to avoid cleaning sampling sites or using cosmetics for at least 48 h before sampling. A strict procedure of superficial skin sampling was conducted for both cheeks (2 cm × 5 cm) and abdomen (5 cm × 8 cm) in a temperature- (22 ± 2°C) and humidity-controlled (40–60%) room. Samples were collected with sterile cotton-tipped swabs soaked in 0.15 mol/L sodium chloride and 0.1% Tween 20. Each cotton tip was cut, placed in a 2.0-ml sterile centrifuge tube, and frozen in liquid nitrogen immediately for 5 min, then stored at −80°C until DNA extraction.

### Facial Skin Parameters Collection

The VISIA Complexion Analysis System (Canfield, Fairfield, NJ, United States) was used to take photographic images of participants to analyze the physiological characteristics of skin aging, quantitatively. Participants were asked to wash their face with clean water before this examination. Images were taken from the front and 45° to both lateral under the same conditions to ensure the sampling sites (both cheeks) were covered completely. The photographic images were of 10-megapixel resolution and were captured with standard, cross-polarized, parallel polarized, and UV light. Spots, wrinkles, texture, pores, UV spots, brown spots, red areas, and porphyrins indexes were all objectively evaluated by the system. Absolute scores were used to assess the actual condition of each evaluation index.

### DNA Extraction, PCR Amplification, and Sequencing

Total genomic DNA was extracted from the clipped swabs with the PowerSoil DNA isolation kit (MoBio Laboratories, Carlsbad, CA, United States) according to the manufacturer’s instructions. DNA concentration was measured with a NanoDrop spectrophotometer (Thermo Fisher Scientific). Hypervariable regions V3–V4 of the bacterial 16S rDNA were amplified with the primers 338F (ACTCCTACGGGAGGCAGCAG) and 806R (GGACTACHVGGGTWTCTAAT) (Munyaka et al., 2015). The fungal ITS region was amplified with the primers ITS1F (5-GGAAGTAAAAGTCGTAACAAGG-3) and ITS2 (5-TCCTCCGCTTATTGATATGC-3) (Zhang et al., 2015). Amplification was performed as described by Cao et al. (2018). The PCR products were purified and normalized using Agencourt AMPure XP magnetic beads (Beckman Coulter) according to the manufacturer’s protocol. Purified amplifications were quantitated with real-time PCR and pooled for Illumina sequencing. Deep sequencing was performed 2 × 300-bp paired-end on an Illumina MiSeq platform using MiSeq Reagent Kit v3 (600 cycles) (Illumina) according to the standard protocol.

### Real-Time Quantitative PCR

Total DNA was isolated with AxyPrep Multisource Genomic DNA Kit (Axygen, Union City, CA, United States) according to the manufacturer’s instructions, and DNA purity was checked through the NanoPhotometer spectrophotometer (IMPLEN, Westlake Village, CA, United States). Microbial genera of *Staphylococcus, Cutibacterium, Lactobacillus*, and *Corynebacterium* were quantified by real-time quantitative PCR (qPCR) in different groups. The genus-specific primer pairs used for qPCR are shown in Supplementary Table 5 (Martineau et al., 2001; Rinttilä et al., 2004). The detailed protocol of the qPCR is shown in the Supplementary Protocol 1. Data were analyzed with ABI 7500 Real-Time PCR software version 2.0.5, and we used the second derivate maximum method, which calculated PCR efficiency in accordance with Pfaffl (2001). Sample DNA concentration was calculated by comparing the sample Cp value to the standard curve. Each group was tested in triplicate.

### Taxonomic Unit Clustering and Assignment

We assigned all reads to each specific sample on the basis of the barcode sequence. Reads were removed if they were < 200 bp, with an average low-quality score (≤20), containing ambiguous bases or not exactly matched to the universal primer. For chimera checking and taxonomy assignment of similar high-quality sequences, qualified reads were *de novo* clustered into operational taxonomic units (OTUs) at a similarity level of 97% (Edgar, 2013), and chimeric OTUs were identified by the UCHIME algorithm (v1.1.3) and removed. The Ribosomal Database Project Classifier tool was used to classify all qualified sequences into different taxonomic groups (Cole et al., 2009).

The taxonomy of each 16S rDNA and ITS rDNA sequence was analyzed by UCLUST with the corresponding Silva 16S rRNA database and Unite database, respectively. Chao1-estimated OTU number index was also used to assess species diversity. Beta diversity was evaluated by calculating UniFrac distance (weighted) and constructing from the OTU table principal component analysis between the samples in different groups. Spearman analysis was used to analyze the correlation between clinical data and dominant microbiomes, and the *P-value* was calculated with Mothur software, an OTU association program. The bacterial metabolic potentials were predicted by Tax4FUN software combined with the Kyoto Encyclopedia of Genes and Genomes (KEGG) database. FUNGuid software was used to predict fungal functions. All raw sequencing reads analyzed were deposited in the National Center for Biotechnology Information (NCBI) with the accession numbers PRJNA630834 and PRJNA630117.

### Statistical Analysis

According to different data types, various statistical strategies were used to determine statistical significance. Linear discriminant analysis effect size (LEfSe) analysis with a threshold of 3.0 was used to determine the significant microbial taxa among the four age groups. Wilcoxon’s rank-sum test analysis was used to evaluate the statistical differences in the relative abundance of microbial communities, species diversity, and pathway enrichment between the two groups. A Kruskal–Wallis rank-sum test was constructed to estimate the differences in microbial composition and species richness for multiple comparisons. In addition, we also used an analysis of similarities (ANOSIM) based on weighted UniFrac to calculate the differences in community structure. Spearman correlations were used to calculate the relationships between every two microbial genera. All these statistical analyses were performed using R software (v 3.5.0) and mothur software (v1.33.3). *P-value* < 0.05 was considered statistically significant.

## Results

### Clinical Parameters in Facial Skin Aging

We used the VISIA Complexion Analysis System to evaluate the following eight indicators for facial skin from 80 healthy participants in four age groups: spots, wrinkles, texture, pores, UV spots, brown spots, red areas, and porphyrins (see Table 1 and Supplementary Table 6). The absolute scores (ASs) of spots, UV spots, brown spots, red areas, wrinkles, and texture increased with increased age. However, the ASs of pores and porphyrins increased initially and then decreased during aging, and we detected peaks in the middle-aged and youth groups. These eight clinical parameters accurately reflect the changes of skin physiological state in aging in many respects, and they present the regular variation in patterns during aging. Thus, we used these parameters to quantitatively describe the different facial skin aging status and to perform an association analysis of microbiomes.

**TABLE 1:** Clinical skin parameters of cheeks from different age groups.

**Group**	**Children Median (25th, 75th)**	**Youth Median (25th, 75th)**	**Middle age Median (25th, 75th)**	**Elderly Median (25th, 75th)**	***P-*value**
Spots	17.87 (13.78, 21.47)	25.12 (21.07, 27.38)	32.80 (29.81, 35.22)	38.08 (34.97, 44.38)	<0.001
UV spots	5.30 (2.75, 7.94)	11.16 (5.79, 15.23)	11.37 (8.82, 16.57)	14.19 (9.89, 20.01)	<0.001
Brown spots	21.49 (19.43, 25.23)	3016 (25.27, 33.62)	43.53 (37.09, 46.01)	54.49 (51.98, 62.08)	<0.001
Red areas	25.68 (19.91, 29.60)	27.36 (25.35, 33.77)	33.28 (28.72, 34.81)	39.25 (36.84, 42.97)	<0.001
Wrinkles	4.34 (2.80, 7.88)	7.59 (3.73, 16.21)	17.61 (11.65, 22.40)	18.22 (16.60, 23.67)	<0.001
Texture	0.78 (0.40, 1.81)	4.46 (1.50, 5.30)	10.45 (5.51, 14.12)	18.43 (14.76, 24.08)	<0.001
Pores	0.61 (0.51, 0.73)	8.51 (5.97, 13.56)	18.28 (13.85, 21.71)	15.58 (12.40, 16.49)	<0.001
Porphyrins	1.12 (0.65, 1.48)	9.91 (5.12, 15.83)	7.62 (5.62, 10.71)	4.53 (2.04, 6.76)	<0.001

*Median score and quartile (25th percentile, 75th percentile) were used to describe the absolute scores of different indices. P-values were calculated by the Kruskal–Wallis test. Significance level was 0.05.*

### Overall Sequencing of Skin Microbiomes

We extracted DNA from 160 samples of exposed sites (cheek) and non-exposed sites (abdomen) and compiled their rDNA sequences and OTUs (Table 2). We obtained 10,036,756 and 14,864,480 reads from 16S rDNA and ITS rDNA sequencing, respectively. Sequence assembly, chimera removal, and quality filtering resulted in 9,315,093 high-quality 16S rDNA sequences (average 58,219 reads/sample, from 26,245 reads/sample to 154,394 reads/sample) and 14,951,548 high-quality ITS rDNA sequences (average 92,903 reads/sample, from 26,370 reads/sample to 262,051 reads/sample). We clustered these data into 6,813 bacterial and 8,080 fungal OTUs with a ≥ 97% identity threshold. Forty-three phyla and 1,087 genera were annotated with taxonomic information based on bacterial OTUs, and 19 phyla and 975 genera were annotated based on fungal OTUs.

**TABLE 2:** Descriptive information and statistics for each group, i.e., children group (cheek [CCHG], abdomen [ACHG]), youth group (cheek [CYHG], abdomen [AYHG]), middle-aged group (cheek [CMAG], abdomen [AMAG]), and elder group (cheek [CELG], abdomen [AELG]).

**Group**		**Exposed sampling sites**				**Non-exposed sampling sites**		
		**Row tags (Mean ± SD)**	**OTUs (Mean ± SD)**	**Chao 1 (Mean ± SD)**		**Row tags (Mean ± SD)**	**OTUs (Mean ± SD)**	**Chao 1 (Mean ± SD)**
16S rRNA sequencing	CCHG	57,314.95 ± 18,550.61	706.95 ± 142.04	871.00 ± 136.36	ACHG	55,010.35 ± 14,018.03	693.75 ± 150.12	790.94 ± 124.74
	CYHG	57,314.95 ± 18,550.61	765.40 ± 195.63	906.98 ± 160.88	AYHG	52339.65 ± 15,372.78	627.35 ± 69.63	773.09 ± 74.51
	CMAG	68,455.80 ± 18,127.31	736.30 ± 130.98	1002.43 ± 133.11	AMAG	64,877.40 ± 16,541.71	721.55 ± 149.22	977.68 ± 173.76
	CELG	76,821.60 ± 34,202.71	792.80 ± 135.89	1,113.29 ± 154.79	AELG	67,124.55 ± 16,017.87	859.20 ± 214.47	1,132.56 ± 213.50
ITS gene sequencing	CCHG	57,314.98 ± 78,550.61	629.35 ± 144.07	831.37 ± 169.83	ACHG	81,292.35 ± 36,065.16	432.45 ± 157.85	613.31 ± 155.83
	CYHG	59,893.50 ± 15,931.53	665.20 ± 189.05	878.92 ± 250.46	AYHG	77,415.25 ± 44,384.18	474.20 ± 221.25	658.61 ± 315.54
	CMAG	68,455.80 ± 18,127.31	757.55 ± 96.95	1,047.49 ± 139.19	AMAG	95,945.80 ± 51,417.13	772.60 ± 174.85	1,024.11 ± 205.99
	CELG	76,821.60 ± 34,202.71	631.35 ± 271.73	867.09 ± 383.69	AELG	127,038.30 ± 58,757.97	594.80 ± 275.75	843.19 ± 387.64

*Variables are represented as mean ± standard deviation according to row tags, OTUs, and species richness indices (Chao 1 indices).*

### Variation in Patterns of Skin Microbiomes During Intrinsic Aging

**FIGURE 1:**
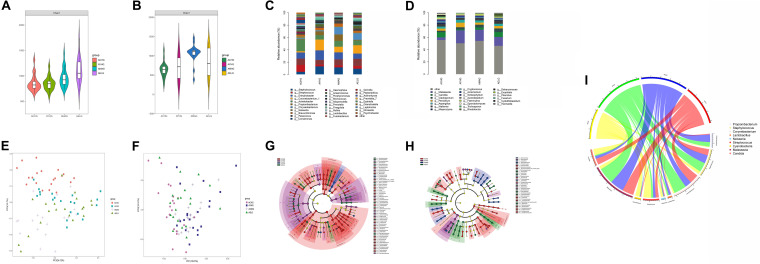
Variation in patterns of skin microbiome during the intrinsic aging process. Bacterial **(A)** and fungal **(B)** species richness at each age group. Relative abundances of bacterial **(C)** and fungal **(D)** core species at the genus level in each group. Weighted UniFrac-PCoA plots of skin bacteria **(E)** and fungi **(F)** in each group. Bacterial **(G)** and fungal **(H)** phylogenetic distributions during intrinsic skin aging (LDA score > 3, *P-*value < 0.05). Based on linear discriminant analysis effect size (LEfSe), the phylogenetic distributions of distinct taxa are colored corresponding to the different classification (phyla to species were depicted by circles from the inside to outside). Circlize analysis shows the relative abundances of nine unique age-related functional taxa in each age group **(I)**.

We detected different composition characteristics of skin microbiomes for the intrinsic aging process for unexposed sites in different aging stages. We found large differences in bacterial and fungal species diversity with Chao1 indices, particularly during the skin intrinsic aging process (Supplementary Table 7). Bacterial species richness increased gradually with advancing age, whereas the fungal communities in the middle-aged group showed the highest species diversity (Figures 1A,B). We further recorded the core species at the phylum and genus levels. Four major bacterial phyla and two fungal core phyla: *Proteobacteria*, *Firmicutes*, *Actinobacteria, Bacteroidetes, Ascomycota*, and *Basidiomycota* were dominant in the four age groups, accounting for > 78% of the total skin microbiomes. In addition, among the top five dominant bacterial species, we detected higher abundances of *Cyanobacteria* in the children group (4.67%) and *Fusobacteria* in the elder group (1.24%) compared with the other age groups. At the genus level, nine dominant bacterial genera (*Streptococcus*, *Porphyromonas*, *Acinetobacter*, *Enhydrobacter*, *Comamonas*, *Staphylococcus*, *Cutibacterium, Corynebacterium*, and *Chryseobacterium*) and eight dominant fungal genera (*Candida*, *Malassezia*, *Penicillium*, *Wallemia*, *Aspergillus*, and *Cladosporium*) were the top five species in at least one group, with average abundances of ≥ 1% (Supplementary Table 1 and Figures 1C,D).

We used weighted UniFrac distance analysis to measure the overall structural similarity and differences in microbiomes associated with skin aging. Significant separations among clusters existed between different age groups with ANOSIM (*P* < 0.05) (Supplementary Table 2). Based on weighted UniFrac-principal coordinates analysis (PCoA), the microbial composition in the children group was obviously different from that in the other age groups. However, the structures of the microbial communities in the other three groups were similar (Figures 1E,F).

We next used LEfSe analysis to compare the differences in taxonomic profiles between the four age groups. At the phylum level, *Proteobacteria* (48.27%), *Cyanobacteria* (4.11%), and *Fusobacteria* (1.80%) were significantly enriched in the children group. In addition, the abundance of *Actinobacteria* (28.18%) was greater in the youth group compared with the other three age groups. Comparative analysis of genera on skin demonstrated that most age-related species predominated in the children group for both bacterial communities, such as *Acinetobacter* (14.33%), *Streptococcus* (8.50%), and *Neisseria* (2.41%), and fungal communities, such as *Trichosporon* (1.12%), *Candida* (6.71%), and *Meyerozyma* (2.18%). We also found significant enrichment of *Staphylococc*us in the youth (10.25%) and middle-aged (8.88%) groups and *Corynebacterium* in the youth (13.84%) group. In addition, *Malassezia* was uniquely more abundant in the youth (17.72%), middle-aged (14.99%), and elder groups (12.37%) (Supplementary Table 3 and Figures 1G,H).

We also performed qPCR for our focused *Staphylococcus* and *Corynebacterium*, closely related to the integrity of the skin immune barrier during skin intrinsic aging. The quantitative results were highly consistent with the results of the 16S rDNA sequencing. The details are shown in Supplementary Figure 2. We believe that the results obtained from the amplicons could well-reflect the real situation of the microbial variation patterns in skin aging processes, and the results are authentic and reliable.

### Variation in Patterns of Skin Microbiomes During Photoaging

**FIGURE 2:**
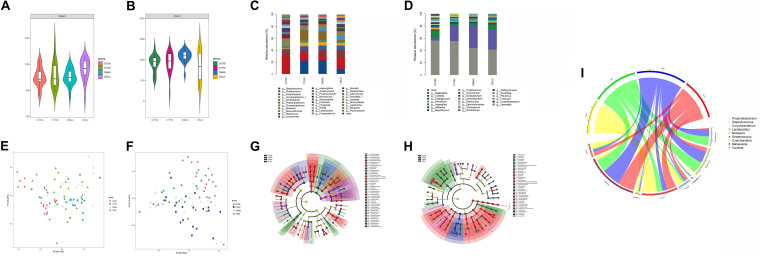
Variation in patterns of skin microbiome during photoaging. Bacterial **(A)** and fungal **(B)** species richness at each age group during photoaging. Relative abundances of bacterial **(C)** and fungal **(D)** core species at the genus level in each group. Weighted UniFrac-PCoA plots of skin bacteria **(E)** and fungi **(F)** in each group. Based on LEfSe, bacterial **(G)** and fungal **(H)** phylogenetic distributions during photoaging (LDA score > 3, *P-*value < 0.05). Circlize analysis shows the relative abundances of nine unique age-related functional taxa in each age group **(I)**.

Due to exogenous factors such as UV radiation, facial skin experiences excessive photoaging. Interestingly, we found that the cheek microbiomes during aging presented unique dynamic variation patterns different from intrinsic aging. The dynamic changes in species richness and core dominant phyla during photoaging were similar to the changes during intrinsic aging. With advancing age, bacterial alpha diversity increased monotonically, whereas the highest species diversity of fungal communities was detected in the middle-aged group (Figures 2A,B). Six major phyla were found by 16S rDNA sequencing, and ITS rDNA sequencing: *Proteobacteria*, *Firmicutes*, *Actinobacteria, Bacteroidetes, Ascomycota*, and *Basidiomycota* was dominant in all age groups. *Cyanobacteria* and *Fusobacteria*on exposed skin were also dominant in the children group and elder group, respectively. For the genera on exposed skin, ten dominant bacterial genera (*Streptococcus*, *Porphyromonas*, *Alloprevotella*, *Enhydrobacter*, *Haemophilus*, *Staphylococcus*, *Cutibacterium, Neisseria, Corynebacterium*, and *Chryseobacterium*) and nine dominant fungal genera (*Candida*, *Malassezia*, *Penicillium*, *Acremonium*, *Exophiala, Meyerozyma, Trichosporon, Aspergillus*, and *Cladosporium*) were the top five dominant genuses in at least one group (Supplementary Table 1 and Figures 2C,D).

When analyzing the beta diversities of the facial skin microbiomes during photoaging, we found that, apart from the similar bacterial composition between the youth and middle-aged groups, significant separations among clusters with ANOSIM (*P* < 0.05) existed between different age groups (Supplementary Table 2). Based on weighted UniFrac-PCoA for both bacterial and fungal profiles, a great difference in microbial composition between children and the other age groups was also present during photoaging. Additionally, a significant difference existed between the youth and elder groups, whereas a similar microbial composition was identified between the middle-aged and both youth and elder groups (Figures 2E,F).

Differential analysis of taxa by LEfSe of the four age groups revealed that, at the phylum level, there was higher enrichment for *Cyanobacteria* in the children group (4.11%), *Actinobacteria* (26.47%) in the youth group, and *Bacteroidetes* (14.56%) in the elder group. Bacterial genus-level assignment similarly showed that more age-related species were dominant in the children group, including *Neisseria* (8.91%), *Streptococcus* (24.35%), and *Haemophilus* (5.15%) and fungal communities such as *Candida* (8.90%) and *Trichosporon* (2.36%). In contrast, *Cutibacterium* (15.13%; 12.12%) and *Staphylococcus* (19.22%; 19.48%) were enriched in both the youth and the middle-aged groups. Compared with the other three age groups, there was a higher proportion of *Lactobacillus* (1.15%) on exposed sites in the middle-aged group. *Streptococcus, Leptotrichia, Alkanindiges*, and *Enhydrobacter* were more likely to reside on cheeks in the elder group. *Malassezia*, the most abundant skin fungus, had a significantly higher proportion in the middle-aged (30.60%) and the elder (27.46%) groups (Supplementary Table 4 and Figures 2G,H). Similarly, quantitative analysis was performed with qPCR for skin immune barrier-related genus during photoaging processes, including *Staphylococcus, Cutibacterium*, and *Lactobacillus*, which were highly consistent with our results of 16S rDNA sequencing (Supplementary Figure 2).

### Relationship Between Clinical Skin Parameters and Microbial Communities in Photoaging

**FIGURE 3:**
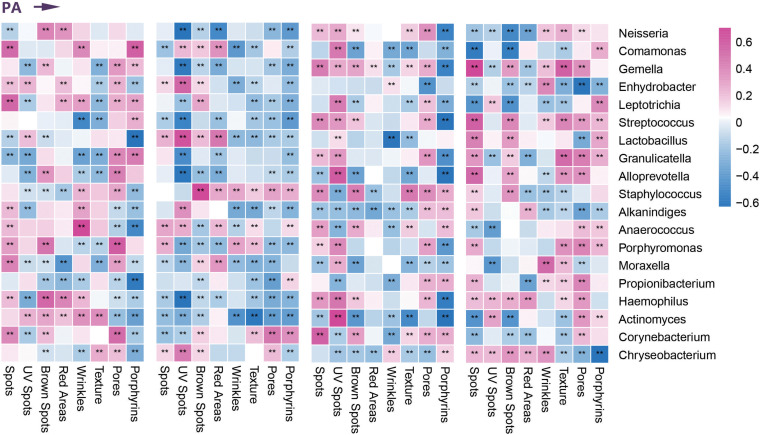
Correlation between clinical parameters and dominant bacterial communities. Heatmap of the Spearman correlation between clinical data and dominant bacterial communities in four age groups during photoaging (PA). **Represents the *p* value <0.01 for the Spearman correlation analysis.

On the basis of differential analysis of taxa of the four age groups, we performed Spearman correlation analysis to describe the relationships between dominant facial microbial communities and age-related clinical parameters (based on the VISIA Complexion Analysis System). We found that the enrichment of *Neisseria* and *Candida* in the children and elder groups correlated positively with the ASs of brown spots and red areas. In the elder group, the higher abundance of *Streptococcus* showed a positive correlation with exacerbation of the progress of emerging (brown) spots, wrinkles, texture, and porphyrins, whereas we observed a nearly opposite trend for the children group. Because enrichment of *Lactobacillus* was present in the middle-aged group, except for UV spots, all remaining indicators declined to varying degrees. Similarly, the normal number of *Cutibacterium* was also considered a protective factor according to the skin parameters. Only the ASs of porphyrins and pores associated with sebum secretion increased with a higher abundance of *Cutibacterium* in the young and middle-aged groups. Most indices indicated a slowing of skin aging. In addition, we found remarkable downward trends in the ASs of UV spots and red areas due to the enrichment of *Staphylococcus* in the young and middle-aged groups. *Malassezia* was the most dominant fungi on human skin, especially for the middle-aged and elder groups. Most of the parameters suggested that skin was more likely to be in a senescent state when abundant *Malassezia* was present. At the phylum level, *Cyanobacteria* was a newly detected predominant phylum in the children group related to preventing UV-induced damage and pigmentation, including spots, UV spots, and brown spots (Figure 3 and Supplementary Figure 1). We ultimately identified nine microbial communities most closely associated with age-related clinical parameters and demonstrated their relative abundance in each age group in Figures 1I, 2I.

### Functional Prediction of Skin Microbiomes in Skin Aging

**FIGURE 4:**
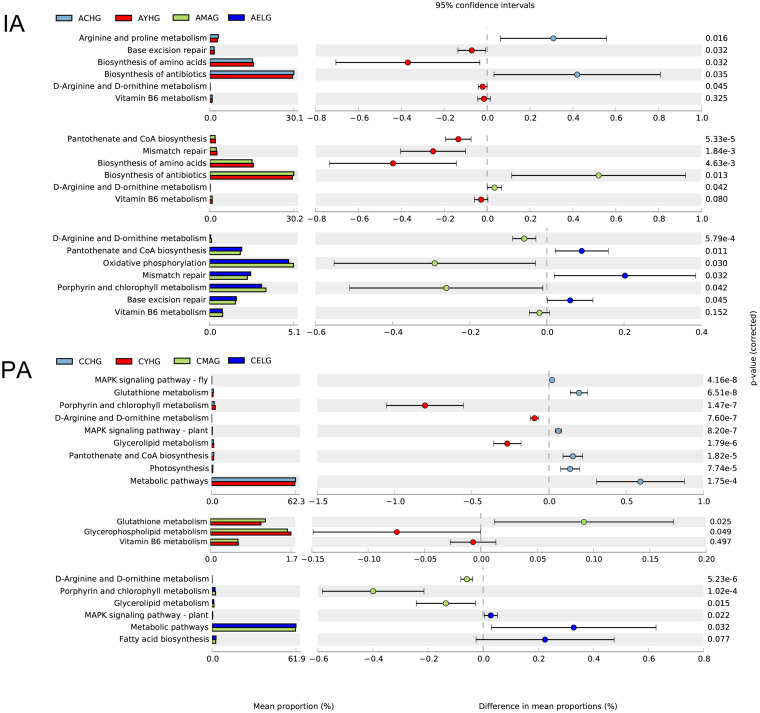
Functional prediction of skin microbiomes in skin aging. Comparison of potential metabolic functions in the Kyoto Encyclopedia of Genes and Genomes pathways between each two age groups during intrinsic skin aging (IA) and photoaging (PA).

Apparently, during intrinsic aging, different modes of action of skin microbiomes occur in different stages. During the transition from children to youth, we found higher enrichments in *base excision repair*, *biosynthesis of amino acids*, and *biosynthesis of antibiotics* and lower abundances of *arginine* and *proline metabolism* in the youth group. With the progression of aging, *pantothenate and CoA biosynthesis* and *mismatch repair* pathways gradually lost their dominance in the middle-aged group, and lower enrichment in *D-arginine and D-ornithine metabolism* and *oxidative phosphorylation* in the elder group may contribute to the intrinsic aging process. Remarkably, the abundance of *vitamin B6 metabolism* continually declined from youth to elder (Figure 4).

During cheek photoaging, microbial alterations affect the aging process from children to youth, mainly by downregulating the *MAPK signaling pathway*, *glutathione metabolism*, *photosynthesis*, and *pantothenate and CoA biosynthesis*. In the subsequent aging stage, microbiomes in the middle-aged group decrease their metabolic capacities, such as *glycerophospholipid metabolism*, *vitamin B6 metabolism*, and *metabolic pathways*. We found that pathways associated with biosynthesis and lipid metabolism, i.e., *biosynthesis of antibiotics*, *fatty acid biosynthesis*, *glycerol lipid metabolism*, and *porphyrin and chlorophyll metabolism* gradually lost dominance in the elder group (Figure 4).

## Discussion

Physiological skin changes are thought to mirror aging. Because skin aging is prone to be affected by environmental factors, especially UV radiation, previous studies distinguished between intrinsic aging and photoaging (Bonté et al., 2019; Wang et al., 2019). Not only is skin aging associated with a decline in appearance and the occurrence of skin disorders, but also the bacterial composition of facial skin among women was strongly affected by aging (Makrantonaki et al., 2012; Blume-Peytavi et al., 2016; Shibagaki et al., 2017; Kim et al., 2019). In our research, for the first time, we comprehensively revealed the various age-dependent patterns in the composition of skin prokaryotic and eukaryotic communities. We also suggest that our findings represent a new stage in the understanding of the skin pan-microbiomes and characteristics of skin aging.

### Similar Microbial Variation Patterns in Intrinsic Skin Aging and Photoaging

In aging, we detected different species diversities and compositional characteristics of skin microbiomes in different age groups. These differences could be ascribed to continuous self-regulation and self-renewal of skin microbiomes, related to individual’s residential environment and host selection pressures (e.g., local physiological status) (Kim et al., 2018; Dimitriu et al., 2019). Consistent with previous studies, higher species diversities of cutaneous bacterial communities were found with aging for both exposed and non-exposed sites (Leung et al., 2015, 2016a; Shibagaki et al., 2017). However, we found that, for both sampling sites, the fungal communities showed the highest species diversity in the middle-aged group (Figures 1A,B, 2A,B). In addition, we noted that the most conspicuous structural differences were found between the children and the other age groups (Figures 1E, 2E). With the onset of puberty, transition, and sexual maturation, the skin microbiomes would undergo a large shift, with the disappearance of several taxa. Because sex hormones stimulate remodeling of the local sebaceous environment, microorganisms become dominated by lipophilic communities such as *Cutibacterium* and *Malassezia* in the middle-aged and elder groups (Leyden et al., 1975; Oh et al., 2012; Belkaid and Tamoutounour, 2016).

**FIGURE 5:**
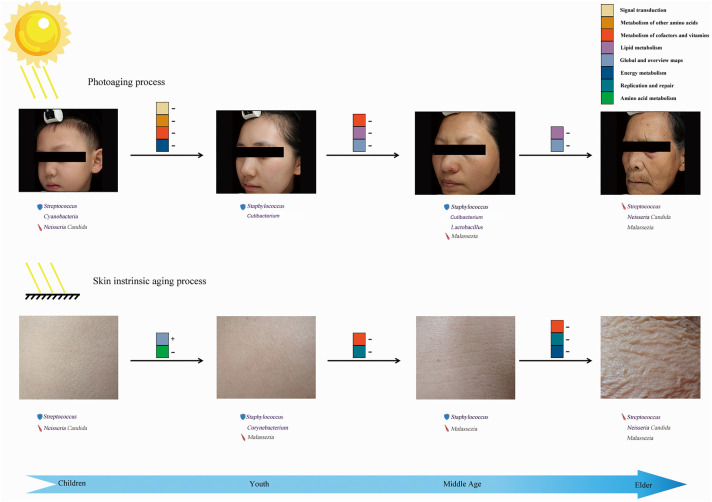
Overview of microbial action mode in skin aging. The “shield” before the microbial communities represents the protective factors, whereas the “sword” could be treated as unfavorable factors during skin aging. “+” followed by aging indicating potential functional pathways means that the abundance of a pathway increased with aging, whereas “–” indicates an opposite trend for the variation.

We further identified several potentially functional skin age-related microbiotas combined with quantitative analysis. By exploring the specific distribution of these dominant communities and the proved functions associated with clinical characteristics, we hypothesized that microbial communities not only coexist with the skin barrier but might actually modify the process of skin aging, as proposed by current theories (Roth and James, 1988; Sanford and Gallo, 2013). Because diverse microbial compositions exist in different sites and different age groups, the potential effects of microorganisms on the skin are age- and site-specific (Figure 5). After identifying the nine microbial communities closely related to skin aging parameters, the potential relationships between these genera (including bacteria and fungi) were also expounded in the aging process (Supplementary Figure 3). Furthermore, our work also mainly focused on the effect of aging on the changes in abundance of certain genera and compositional contribution of the entire microbiomes, rather than the effects of aging on the changes of overall abundance or absolute quantity of microbiomes.

### Specific Age-Related Microbial Communities and Pathways to Regulate Skin Photoaging

We observed a higher abundance of *Cyanobacteria* in the children group. As vital components of the photoprotective barrier, *Cyanobacteria* have developed protective mechanisms against ultraviolet radiation on the skin (Fuentes-Tristan et al., 2019). In correlation analysis with clinical data, enrichment of *Cyanobacteria* was greatly related to decreased UV-induced damage and pigmentation. In addition, significant enrichment of photosynthesis-related pathways indicated that *Cyanobacteria* were more active and had a more important function in the children group. On the basis of this evidence, we speculate that children should have a more complete anti-UV barrier.

A previous study showed that the skin aging process could be regulated and even reversed by restoring the normal barrier functions (An et al., 2017), and, during photoaging, the immune–microbial alliance is considered a bridge between innate and adaptive immunity to preserve barrier integrity by microbiota. Association analysis with clinical parameters revealed that the specific enrichment of *Staphylococcus, Cutibacterium*, and *Lactobacillus* benefited some detected indicators (e.g., UV spots, red areas), and we believed that these microbiotas were likely to improve the skin barrier integrity. In the youth or middle-aged groups, higher enrichment of *Staphylococcus* could produce several antimicrobial proteins and proteases that protect the skin from pathogenic invasion and maintain skin microbiota homeostasis (Gallo and Hooper, 2012; Buffie and Pamer, 2013; Belkaid and Tamoutounour, 2016). Meanwhile, *Cutibacterium* could mediate the necessary immune response and suppress inflammation to further slow the aging process by modulating conjugated linoleic acid generation and control the expression of antimicrobial proteins by *Staphylococcus* (Liavonchanka et al., 2006). In the middle-aged group, the higher abundance of *Lactobacillus* may develop protective effects from aging for the following reasons. *Lactobacillus* can produce various antimicrobial substances and induce anti-inflammatory Treg cells to reduce inflammatory injury caused by UV radiation (Oh et al., 2006), potentially improving conditions for the delay in collagen synthesis and wrinkle formation (Landen et al., 2016), consistent with our skin parameters. The mentioned microbial functions are the specific and inherent characteristics of these three microbiotas, which could well-explain the results we obtained. Additionally, *Lactobacillus* could also act synergistically with *Staphylococcus*, which likely leads to enrichment in the *biosynthesis of antibiotics*, specifically in the middle-aged group, consistent with the result of the predicted functional pathway. Therefore, in the youth and middle-aged groups, we speculate that *Staphylococcus, Cutibacterium*, and *Lactobacillus* could promote the formation of mature and complete immune barriers or host defense, to further protect during photoaging.

On the basis of correlation analysis with the parameters, we regarded *Streptococcus* as a decelerating factor for skin aging in the children and youth groups, whereas an opposite behavior was observed in the other two groups. This interesting finding is potentially attributable to the alteration with the aging of the dominant species *Streptococcus*, from probiotic communities to the conditional pathogenic genera. Because the skin barriers of the children and elder groups exist in an immature and recessive status, respectively, we detected a higher abundance of some conditional pathogenic genera, such as *Neisseria, Streptococcus* (elder group), and *Candida* (Petry et al., 2012; Beatrous et al., 2017; Metin et al., 2018). According to the clinical parameters, these species could be regarded as accelerators (negative correlations) of aging. Due to long-term inflammatory stimulation, even association with various dermatological disorders, high enrichment of *Malassezia* could be considered an unfavorable factor, particularly in the middle-aged and elder groups according to clinical parameters. The analysis of the correlations between selected genera in the process of photoaging confirmed our inference, to some extent (Supplementary Figure 3).

We further depicted specific functional pathways exerted by the entire microbial community at different aging stages (aging-related landscape) with functional prediction analysis. The photoaging process is characterized mainly by variation of metabolic capacity. Due to higher hormone secretion, greater enrichment for pathways related to metabolism was observed in the youth compared with the middle-aged group, which indicated less protective effects of antioxidants (*metabolism of cofactors and vitamins*) (Martínez-Navarro et al., 2020) and decreased ability to provide energy, membrane integrity, and cell signaling (*glycerophospholipid metabolism*) (Cruickshank-Quinn et al., 2017) in the middle-aged group. As aging progresses, in the transition between the middle-aged and the elder groups, further reduction in lipid metabolism capacity (*glycerolipid metabolism*, *fatty acid biosynthesis*) (De Luca and Valacchi, 2010; Cruickshank-Quinn et al., 2017) and resistance to pathogens (*biosynthesis of antibiotics*) jointly promote photoaging. Interestingly, the bacterial communities have two nearly opposite effects on the skin at the children age. Decreasing antioxidant defense substances (*glutathione metabolism*) (Wolfle et al., 2014), epidermal homeostasis (*pantothenate and CoA biosynthesis*) (Kuehne et al., 2017), and UV-protection (*photosynthesis*) are considered unfavorable factors during skin aging. Conversely, less enrichment in the MAPK signaling pathway in the youth group could slow down the photoaging speed, with less degradation of the extracellular matrix (Shin et al., 2019). These predicted functions would help us to better understand the function of the whole skin microbiomes in the photoaging process. Although they cannot be the same as the actual situation, combined with the previous analysis, we can still obtain meaningful inferences.

### Specific Age-Related Microbial Communities and Pathways to Regulate Intrinsic Skin Aging

Intrinsic skin aging is associated with the variation of immune homeostasis and defective barrier function (Zeng et al., 2019), so the immune–microbial alliance is also a vital factor that regulates the intrinsic aging process. We found significant enrichment of *Staphylococc*us in the youth and middle-aged groups, which would potentially maintain the integrity and healthy status of the skin immune barrier to further affect intrinsic aging. We also speculate that *Corynebacterium* is another age-related anti-aging factor, particularly *Corynebacterium jeikeium* (a resident bacteria on the skin), which usually produces several bacteriocin-like compounds against pathogens. Furthermore, the ability of manganese acquisition reduces oxidative damage to the epidermal tissue of non-exposed sites (Storz and Imlay, 1999; Cogen et al., 2008). In the youth group, according to the microbial inherent characteristics, *Corynebacterium* would act synergistically with *Staphylococcus*, where it would contribute to the enrichment of the *biosynthesis of antibiotics.* Because of the instability of the skin barrier in the children and elder groups, a higher proportion of pathogenic genera such as *Neisseria*, *Streptococcus* (elder group), and *Candida* are also regarded as unfavorable factors during intrinsic aging. Besides, we especially detected higher enrichment of *Malassezia* in the youth, middle-aged, and elder groups, which could be related to the long-term inflammatory stimulation to accelerate aging. The selected intrinsic skin aging-related six bacteria and fungi had significant correlations and relative variation patterns, as demonstrated in Supplementary Figure 3, which also could support the potential interactions between different microbial communities (including bacteria and fungi). We also found that, by the enrichment of specific signaling pathways, bacterial communities have different activities in the intrinsic process of skin aging.

The variations of predicted functions were also discussed in intrinsic aging progress. Sustained damage of DNA repair (*mismatch repair, base excision repair*) and antioxidant capacity (*metabolism of cofactors and vitamins*) during the youth to elder stages would accelerate intrinsic aging. In addition, compared with the middle-aged group, epidermal tissue is in a more healthy and stable state in the youth group (*pantothenate and CoA biosynthesis*) (Kuehne et al., 2017). In the elder group, a reduced energy supply to the host was suggested by the alteration of abundance in *oxidative phosphorylation*. The growth of children into adults is accompanied by increased amino acids synthesis (*biosynthesis of amino acids*) and resistance to pathogens (*biosynthesis of antibiotics*) and by decreased collagen synthesis (arginine and proline metabolism) (Kang et al., 2015) that is associated with facial wrinkles and texture. Therefore, significant differences in the variation of predicted functions for the skin microbiomes were present in the two aging processes. According to the functional prediction analysis, we found that photoaging was accompanied mainly by decreasing the biosynthesis and metabolism of age-related functional substances, whereas intrinsic skin aging was characterized by variation in the biological process.

### Limitations

Our study was limited mainly to function prediction analysis. In this section, we performed the function prediction analysis with Tax4fun software based on the database, which cannot be completely consistent with the actual function of the whole skin microbiomes. In addition, we just constructed the relationships between predicted results and potentially functionally dominant genera, whereas their real association cannot be determined. Therefore, additional metagenomic studies are needed to confirm our findings. In addition, we selected the cheek and abdomen as representative sites for study, although the microbial compositions in other body parts may be different. Based on fungal functional annotation and current microbiome research, skin fungal communities are recognized mainly as pathogens. More confirmed functional pathways related to microbiome during aging need experimental verification. Although we controlled the sampling process and area strictly, we still might obtain the different total population size for skin microbiomes of each sample. Therefore, the biological repetitions of multiple samples were conducted in each group. The results of qPCR were used to support our current results of relative abundance, to further ensure the reliability of our findings.

## Conclusion

Our study was the first to systematically describe an entire landscape of skin age-related bacterial and fungal profiles (from children to elderly). Due to the changes in individual residential states of skin microbiomes during aging, specific age-related microbial compositions were presented in different age groups. Similar variation patterns of species diversity and compositional structures based on weighted UniFrac-PCoA were detected between intrinsic skin aging and photoaging. Conversely, we identified aging specific dominant microbes and predicted functional pathways for exposed and unexposed sites in each group (Figure 5). We found these potential age-related microbial factors in each group that were likely to affect the integrity of the skin immune and anti-UV barrier, biosynthesis and metabolism of age-related functional substances, and long-term inflammatory stimulation, to further regulate skin intrinsic aging and photoaging processes. Our work provides a new perspective on skin aging, which could be used to develop anti-aging cosmetics and skincare for different ages.

Our work provides a new strategy for future research and clinical application of skin microbiomes. First, by further expanding the research cohort and reducing age stratification, we can describe the variation patterns of skin microbial composition in the aging process more accurately. Then, some specific microbiota can be used as a biomarker to further evaluate skin aging status as a non-invasive auxiliary test. Second, further purification of specific anti-aging microbiota and related metabolites would promote the development of the products related to cosmetic medicine. In addition, based on our findings and further study, skin homeostasis regulators for different age groups can be developed to reduce the incidence of age-related skin diseases. Therefore, our research has great innovation and clinical value.

## Data Availability Statement

The datasets presented in this study can be found in online repositories. The names of the repository/repositories and accession number(s) can be found below: https://www.ncbi.nlm.nih.gov/, PRJNA630834; https://www.ncbi.nlm.nih.gov/, PRJNA630117.

## Ethics Statement

Ethical review and approval was not required for the study on human participants in accordance with the local legislation and institutional requirements. Written informed consent to participate in this study was provided by the participants’ legal guardian/next of kin. Written informed consent was obtained from the individual(s), and minor(s)’ legal guardian/next of kin, for the publication of any potentially identifiable images or data included in this article.

## Author Contributions

DH, LS, ZZ, and ZL contributed to the study and experimental design. ZL, LL, and JY contributed to microbial sample collection. ZL, XY, and JL contributed to the 16S rDNA sequencing data analysis. ZL, YW, TH, and XW contributed to the ITS rDNA sequencing data analysis. ZL, HZ, HW, and KT contributed to the skin parameter collection. ZL and XB contributed to the microbial data analysis and statistics. ZL and TP contributed to the additional data analysis. ZL, XB, and TP wrote the manuscript. All authors read and approved the final manuscript.

## Conflict of Interest

The authors declare that the research was conducted in the absence of any commercial or financial relationships that could be construed as a potential conflict of interest.

## Supplementary Material

Supplementary Figure 1 Correlation between clinical parameters and dominant fungal communities.

Supplementary Figure 2 The bar charts of DNA quantitative results according to qPCR.

Supplementary Figure 3 The line charts and correlations of selected microbiotas.

Supplementary Table 1 Relative abundance of dominant species.

Supplementary Table 2 Comparing the similarity of skin microbial compositions and structures between different age groups.

Supplementary Table 3 Differences in taxonomic profiles of skin microbiomes between age groups during intrinsic skin aging.

Supplementary Table 4 Differences in taxonomic profiles of skin microbiomes between age groups during photoaging.

Supplementary Table 5 The selected genus-specific primer pairs in our study.

Supplementary Table 6 Multitest correction for the statistics (*P*-value) for clinical skin parameters of cheeks.

Supplementary Table 7 Multitest correction for the statistics (*P*-value) for species richness indices (Chao 1 indices).

Supplementary Table 8 Multitest correction for the statistics (*P*-value) for Spearman correlation analysis between skin photoaging related dominant genera.

Supplementary Table 9 Multitest correction for the statistics (*P*-value) for Spearman correlation analysis between skin intrinsic aging related dominant genera.

Supplementary Protocol 1 The protocol of Real-Time Quantitative PCR (qPCR).
