# Supplementary figures and images for: New Insights Into the Skin Microbial Communities and Skin Aging
Source: Front Microbiol. 2020 Oct 26;11:565549. doi: 10.3389/fmicb.2020.565549 (PMC7649423; doi:10.3389/fmicb.2020.565549)

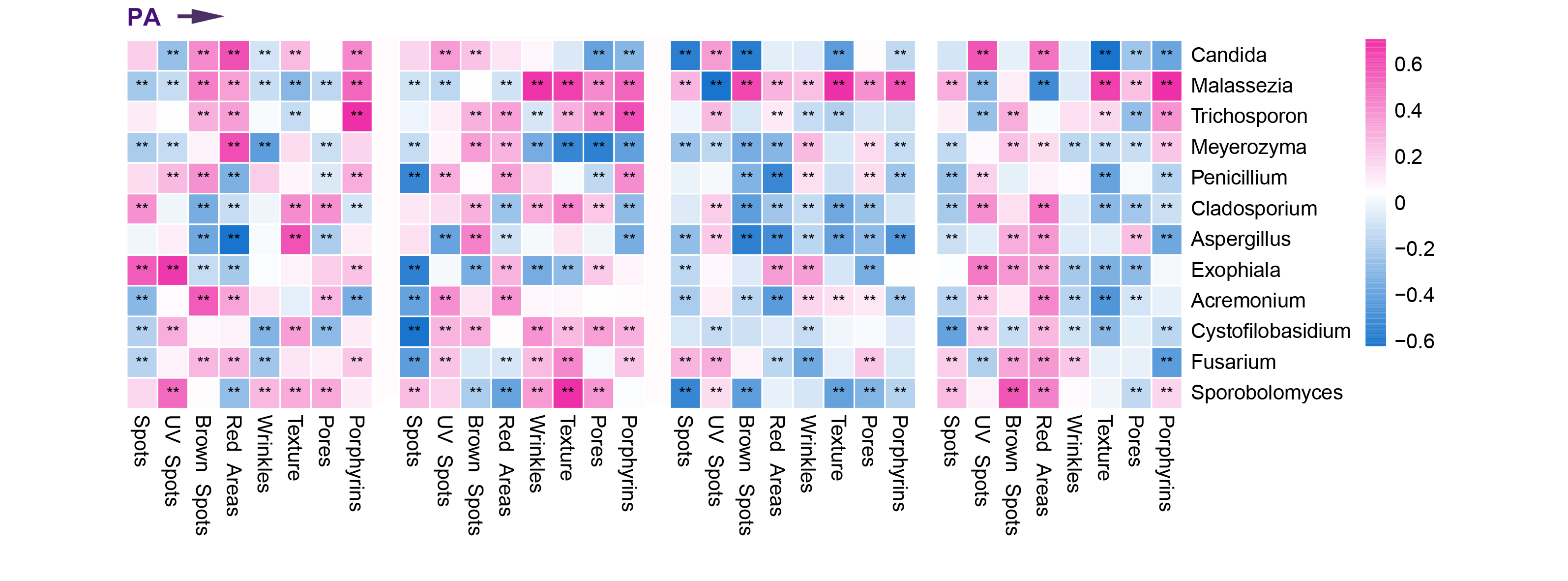

Supplement: Supplementary Figure 1 — Correlation between clinical parameters and dominant fungal communities. [file Image_1.JPEG]

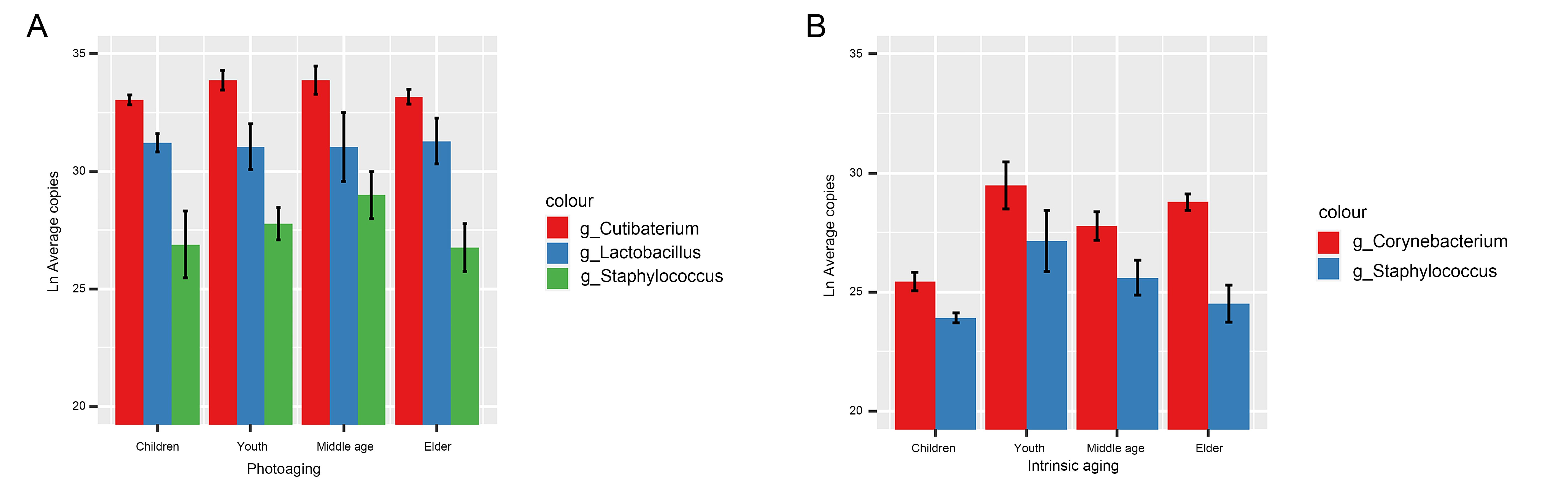

Supplement: Supplementary Figure 2 — The bar charts of DNA quantitative results according to qPCR. [file Image_2.JPEG]

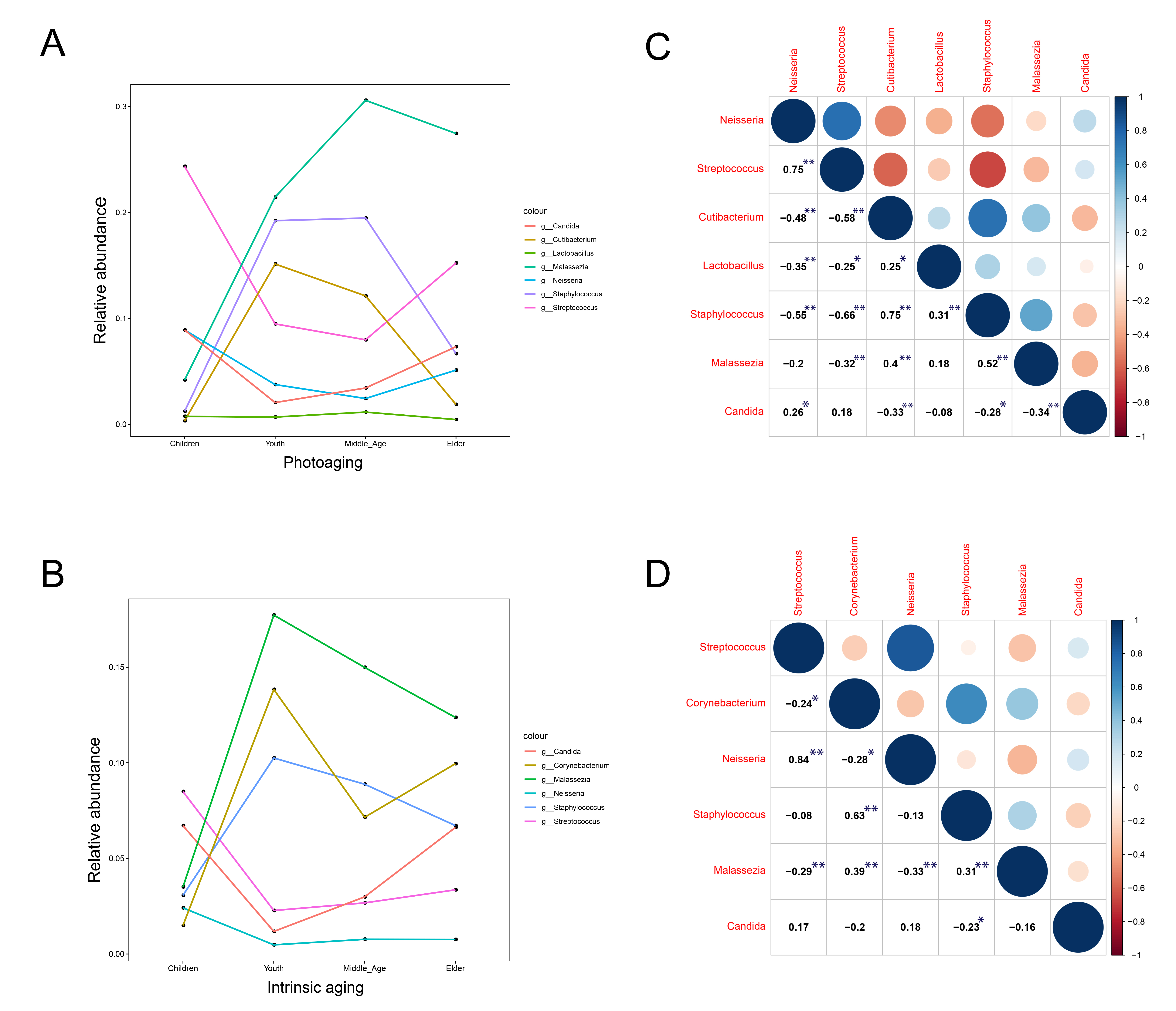

Supplement: Supplementary Figure 3 — The line charts and correlations of selected microbiotas. [file Image_3.JPEG]
