# Supplementary material for: New Insights Into the Skin Microbial Communities and Skin Aging
Source: Front Microbiol. 2020 Oct 26;11:565549. doi: 10.3389/fmicb.2020.565549 (PMC7649423; doi:10.3389/fmicb.2020.565549)
Supplement: Supplementary Table 1 — Relative abundance of dominant species. [file Table_1.DOCX]

|  | Bacterial Phylum | | Bacterial Genus | | Fungal Phylum | | Fungal Genus | |
| --- | --- | --- | --- | --- | --- | --- | --- | --- |
|  | Species | relative abundance | Species | relative abundance | Species | relative abundance | Species | relative abundance |
| CCHG | *Firmicutes* | 38.35% | *Streptococcus* | 24.35% | *Ascomycota* | 53.06% | *Candida* | 8.90% |
|  | *Proteobacteria* | 32.72% | *Neisseria* | 8.91% | *Basidiomycota* | 26.26% | *Malassezia* | 4.19% |
|  | *Bacteroidetes* | 12.51% | *Haemophilus* | 5.15% | *Mortierellomycota* | 0.18% | *Trichosporon* | 2.36% |
|  | *Actinobacteria* | 7.78% | *Alloprevotella* | 4.22% | *Glomeromycota* | 0.13% | *Meyerozyma* | 1.96% |
|  | *Cyanobacteria* | 4.11% | *Porphyromonas* | 2.98% | *Chytridiomycota* | 0.05% | *Penicillium* | 1.85% |
| ACHG | *Proteobacteria* | 48.27% | *Acinetobacter* | 14.33% | *Ascomycota* | 50.36% | *Candida* | 6.71% |
|  | *Firmicutes* | 26.61% | *Streptococcus* | 8.50% | *Basidiomycota* | 27.29% | *Malassezia* | 3.51% |
|  | *Actinobacteria* | 9.78% | *Enhydrobacter* | 7.60% | *Chytridiomycota* | 0.96% | *Wallemia* | 3.36% |
|  | *Bacteroidetes* | 7.77% | *Comamonas* | 4.43% | *Glomeromycota* | 0.62% | *Penicillium* | 2.81% |
|  | *Cyanobacteria* | 4.67% | *Staphylococcus* | 3.07% | *Mortierellomycota* | 0.23% | *Aspergillus* | 2.66% |
| CYHG | *Firmicutes* | 37.89% | *Staphylococcus* | 19.22% | *Ascomycota* | 49.68% | *Malassezia* | 21.45% |
|  | *Actinobacteria* | 26.47% | *Propionibacterium* | 15.13% | *Basidiomycota* | 33.24% | *Cladosporium* | 3.28% |
|  | *Proteobacteria* | 24.83% | *Streptococcus* | 9.48% | *Mortierellomycota* | 0.25% | *Aspergillus* | 2.95% |
|  | *Bacteroidetes* | 7.64% | *Corynebacterium* | 5.13% | *Glomeromycota* | 0.15% | *Candida* | 2.05% |
|  | *Fusobacteria* | 1.17% | *Enhydrobacter* | 4.55% | *Chytridiomycota* | 0.13% | *Exophiala* | 1.30% |
| AYHG | *Proteobacteria* | 32.60% | *Corynebacterium* | 13.84% | *Ascomycota* | 46.96% | *Malassezia* | 17.72% |
|  | *Firmicutes* | 30.95% | *Enhydrobacter* | 12.30% | *Basidiomycota* | 34.27% | *Penicillium* | 6.26% |
|  | *Actinobacteria* | 28.18% | *Staphylococcus* | 10.25% | *Mucoromycota* | 0.21% | *Aspergillus* | 3.85% |
|  | *Bacteroidetes* | 7.01% | *Acinetobacter* | 5.69% | *Mortierellomycota* | 0.19% | *Cladosporium* | 3.00% |
|  | *Cyanobacteria* | 0.41% | *Propionibacterium* | 4.91% | *Glomeromycota* | 0.13% | *Wallemia* | 2.48% |
| CMAG | *Firmicutes* | 34.20% | *Staphylococcus* | 19.48% | *Ascomycota* | 46.40% | *Malassezia* | 30.59% |
|  | *Proteobacteria* | 30.36% | *Propionibacterium* | 12.12% | *Basidiomycota* | 31.96% | *Candida* | 3.43% |
|  | *Actinobacteria* | 22.25% | *Streptococcus* | 7.98% | *Glomeromycota* | 0.22% | *Cladosporium* | 2.40% |
|  | *Bacteroidetes* | 9.84% | *Chryseobacterium* | 5.69% | *Mortierellomycota* | 0.17% | *Aspergillus* | 2.20% |
|  | *Cyanobacteria* | 1.44% | *Enhydrobacter* | 5.58% | *Rozellomycota* | 0.09% | *Acremonium* | 1.32% |
| AMAG | *Proteobacteria* | 38.07% | *Chryseobacterium* | 9.29% | *Ascomycota* | 46.40% | *Malassezia* | 14.99% |
|  | *Actinobacteria* | 24.34% | *Enhydrobacter* | 8.92% | *Basidiomycota* | 31.96% | *Cladosporium* | 3.77% |
|  | *Firmicutes* | 21.86% | *Staphylococcus* | 8.88% | *Glomeromycota* | 0.22% | *Wallemia* | 3.06% |
|  | *Bacteroidetes* | 13.39% | *Propionibacterium* | 8.14% | *Mortierellomycota* | 0.17% | *Candida* | 2.99% |
|  | *Cyanobacteria* | 0.68% | *Acinetobacter* | 7.48% | *Rozellomycota* | 0.09% | *Aspergillus* | 2.85% |
| CELG | *Proteobacteria* | 34.21% | *Streptococcus* | 15.23% | *Ascomycota* | 49.81% | *Malassezia* | 27.46% |
|  | *Firmicutes* | 31.87% | *Staphylococcus* | 6.67% | *Basidiomycota* | 37.45% | *Candida* | 7.32% |
|  | *Bacteroidetes* | 14.56% | *Enhydrobacter* | 5.60% | *Mortierellomycota* | 0.23% | *Cladosporium* | 2.76% |
|  | *Actinobacteria* | 13.30% | *Chryseobacterium* | 5.25% | *Glomeromycota* | 0.16% | *Penicillium* | 2.23% |
|  | *Fusobacteria* | 3.57% | *Neisseria* | 5.11% | *Rozellomycota* | 0.03% | *Aspergillus* | 2.05% |
| AELG | *Proteobacteria* | 31.91% | *Corynebacterium* | 9.95% | *Ascomycota* | 49.16% | *Malassezia* | 12.37% |
|  | *Firmicutes* | 26.42% | *Chryseobacterium* | 7.78% | *Basidiomycota* | 31.87% | *Candida* | 6.62% |
|  | *Actinobacteria* | 24.45% | *Staphylococcus* | 6.71% | *Chytridiomycota* | 0.59% | *Cladosporium* | 5.19% |
|  | *Bacteroidetes* | 13.73% | *Enhydrobacter* | 6.16% | *Glomeromycota* | 0.18% | *Wallemia* | 4.47% |
|  | *Fusobacteria* | 1.24% | *Acinetobacter* | 5.63% | *Mortierellomycota* | 0.04% | *Penicillium* | 3.37% |

Supplementary Table 1 Relative abundance of dominant species. The species and relative abundance of the 5 most abundant phylum and genus of bacterial and fungal communities in each group.
