# Supplementary material for: New Insights Into the Skin Microbial Communities and Skin Aging
Source: Front Microbiol. 2020 Oct 26;11:565549. doi: 10.3389/fmicb.2020.565549 (PMC7649423; doi:10.3389/fmicb.2020.565549)
Supplement: Supplementary Table 2 — Comparing the similarity of skin microbial compositions and structures between different age groups. [file Table_2.DOCX]

| Group | 16S rRNA sequencing | | ITS gene sequencing | |
| --- | --- | --- | --- | --- |
|  | *R value* | *P value* | *R value* | *P value* |
| ACHG-AMAG | 0.486 | 0.001 | 0.2461 | 0.001 |
| ACHG-AELG | 0.3577 | 0.001 | 0.2083 | 0.001 |
| ACHG-AYHG | 0.5353 | 0.001 | 0.2572 | 0.001 |
| AMAG-AELG | 0.1157 | 0.002 | 0.1373 | 0.001 |
| AMAG-AYHG | 0.1249 | 0.002 | 0.188 | 0.001 |
| AELG-AYHG | 0.1726 | 0.001 | 0.2623 | 0.001 |
| CCHG-CMAG | 0.7687 | 0.001 | 0.412 | 0.001 |
| CCHG-CELG | 0.3397 | 0.001 | 0.3833 | 0.001 |
| CCHG-CYHG | 0.7491 | 0.001 | 0.4025 | 0.001 |
| CMAG-CELG | 0.2864 | 0.001 | 0.0853 | 0.012 |
| CMAG-CYHG | 0.0273 | 0.153 | 0.1233 | 0.003 |
| CELG-CYHG | 0.3718 | 0.001 | 0.236 | 0.001 |

Supplementary Table 2 Comparing the similarity of skin microbial compositions and structures between different age groups. Analysis of similarities (ANOSIM) with weighted UniFrac distances were used to compared the similarity of skin microbial compositions and structures of listed two groups. R values and P values of ANOSIM between each two age groups at two sample sites are shown in the table. P value < 0.05 was considered as statistical significance.
