# Supplementary material for: New Insights Into the Skin Microbial Communities and Skin Aging
Source: Front Microbiol. 2020 Oct 26;11:565549. doi: 10.3389/fmicb.2020.565549 (PMC7649423; doi:10.3389/fmicb.2020.565549)
Supplement: Supplementary Table 3 — Differences in taxonomic profiles of skin microbiomes between age groups during intrinsic skin aging. [file Table_3.DOCX]

| Species | | ACHG | AYHG | AMAG | AELG | LDA | *P value* |
| --- | --- | --- | --- | --- | --- | --- | --- |
| Bacterial Genus | *Neisseria* | 2.41% | 0.48% | 0.77% | 0.76% | 3.97 | <0.001 |
|  | *Acinetobacter* | 14.33% | 5.69% | 7.48% | 5.63% | 4.64 | 0.022 |
|  | *Comamonas* | 4.43% | 0.07% | 0.05% | 0.20% | 4.35 | <0.001 |
|  | *Peptoniphilus* | 1.65% | 1.78% | 0.40% | 1.84% | 3.85 | 0.001 |
|  | *Gemella* | 1.16% | 0.21% | 0.31% | 0.32% | 3.67 | <0.001 |
|  | *Streptococcus* | 8.50% | 2.27% | 2.67% | 3.36% | 4.48 | <0.001 |
|  | *Ezakiella* | 1.58% | 1.32% | 0.19% | 1.65% | 3.84 | <0.001 |
|  | *Corynebacterium* | 1.49% | 13.84% | 7.15% | 9.95% | 4.80 | <0.001 |
|  | *Staphylococcus* | 3.07% | 10.25% | 8.88% | 6.71% | 4.53 | <0.001 |
|  | *Psychrobacter* | 1.40% | 0.04% | 0.07% | 0.07% | 3.91 | <0.001 |
|  | *Haemophilus* | 1.74% | 0.33% | 0.39% | 0.29% | 3.83 | <0.001 |
|  | *Fusobacterium* | 1.24% | 0.17% | 0.32% | 0.35% | 3.71 | 0.001 |
| Bacterial Phylum | *Cyanobacteria* | 4.67% | 0.41% | 0.68% | 0.87% | 4.33 | <0.001 |
|  | *Proteobacteria* | 48.27% | 32.60% | 38.07% | 31.91% | 4.92 | 0.013 |
|  | *Actinobacteria* | 9.78% | 28.18% | 24.34% | 24.45% | 4.98 | <0.001 |
|  | *Fusobacteria* | 1.80% | 0.27% | 0.47% | 1.24% | 3.88 | <0.001 |
| Fungal Genus | *Trichosporon* | 1.12% | 0.46% | 0.16% | 0.18% | 3.70 | 0.012 |
|  | *Byssochlamys* | 1.11% | 0.02% | 0.03% | 0.00% | 3.82 | <0.001 |
|  | *Flammulina* | 0.81% | 0.68% | 1.44% | 0.64% | 3.62 | 0.006 |
|  | *Debaryomyces* | 1.88% | 0.14% | 0.28% | 0.82% | 4.00 | <0.001 |
|  | *Wallemia* | 3.36% | 2.48% | 3.06% | 4.47% | 4.11 | <0.001 |
|  | *Candida* | 6.71% | 1.18% | 2.99% | 6.62% | 4.43 | <0.001 |
|  | *Pleurotus* | 1.03% | 0.21% | 0.39% | 0.42% | 3.59 | 0.023 |
|  | *Malassezia* | 3.51% | 17.72% | 14.99% | 12.37% | 4.85 | <0.001 |
|  | *Rhodotorula* | 0.55% | 0.12% | 0.15% | 2.63% | 3.98 | 0.002 |
|  | *Meyerozyma* | 2.18% | 0.25% | 0.58% | 1.33% | 3.95 | 0.001 |

Supplementary Table 3 Differences in taxonomic profiles of skin microbiomes between age groups during intrinsic skin aging. The LDA score and *P values* are calculated by linear discriminant analysis effect size (LEfSe). The significance level is LDA score> 3, *P value* < 0.05.
