# Supplementary material for: New Insights Into the Skin Microbial Communities and Skin Aging
Source: Front Microbiol. 2020 Oct 26;11:565549. doi: 10.3389/fmicb.2020.565549 (PMC7649423; doi:10.3389/fmicb.2020.565549)
Supplement: Supplementary Table 4 — Differences in taxonomic profiles of skin microbiomes between age groups during photoaging. [file Table_4.DOCX]

| Species | | CCHG | CYHG | CMAG | CELG | LDA | *P value* |
| --- | --- | --- | --- | --- | --- | --- | --- |
| Bacterial Genus | *Neisseria* | 8.91% | 3.75% | 2.43% | 5.11% | 4.46 | <0.001 |
|  | *Comamonas* | 2.68% | 0.04% | 0.03% | 0.08% | 4.10 | <0.001 |
|  | *Gemella* | 2.32% | 0.78% | 0.86% | 1.37% | 3.94 | <0.001 |
|  | *Enhydrobacter* | 2.30% | 4.55% | 5.58% | 5.60% | 4.27 | <0.001 |
|  | *Leptotrichia* | 1.10% | 0.28% | 0.26% | 1.24% | 3.82 | <0.001 |
|  | *Streptococcus* | 24.35% | 9.48% | 7.98% | 15.23% | 4.92 | <0.001 |
|  | *Lactobacillus* | 0.75% | 0.68% | 1.15% | 0.45% | 3.45 | <0.001 |
|  | *Granulicatella* | 1.79% | 0.57% | 0.42% | 0.48% | 3.84 | <0.001 |
|  | *Alloprevotella* | 4.22% | 1.73% | 0.76% | 1.13% | 4.26 | <0.001 |
|  | *Staphylococcus* | 1.22% | 19.22% | 19.48% | 6.67% | 4.95 | <0.001 |
|  | *Alkanindiges* | 0.02% | 0.05% | 0.25% | 2.90% | 3.96 | <0.001 |
|  | *Anaerococcus* | 0.26% | 1.23% | 0.82% | 1.00% | 3.61 | <0.001 |
|  | *Porphyromonas* | 2.98% | 1.08% | 0.81% | 1.77% | 4.06 | <0.001 |
|  | *Moraxella* | 1.96% | 0.15% | 0.06% | 0.17% | 3.98 | <0.001 |
|  | *Propionibacterium* | 0.34% | 15.13% | 12.12% | 1.87% | 4.86 | <0.001 |
|  | *Haemophilus* | 5.15% | 1.83% | 1.69% | 1.53% | 4.27 | <0.001 |
|  | *Actinomyces* | 1.49% | 0.75% | 0.46% | 1.40% | 3.74 | <0.001 |
| Bacterial Phylum | *Cyanobacteria* | 4.11% | 1.12% | 1.44% | 0.80% | 4.10 | 0.035 |
|  | *Bacteroidetes* | 12.51% | 7.64% | 9.84% | 14.56% | 4.60 | 0.003 |
|  | *Actinobacteria* | 7.78% | 26.47% | 22.25% | 13.30% | 4.97 | <0.001 |
| Fungal Genus | *Trichosporon* | 2.36% | 0.43% | 0.09% | 0.13% | 4.17 | <0.001 |
|  | *Cystofilobasidium* | 1.50% | 0.07% | 0.31% | 0.46% | 3.80 | 0.004 |
|  | *Candida* | 8.90% | 2.05% | 3.43% | 7.32% | 4.55 | <0.001 |
|  | *Malassezia* | 4.19% | 21.45% | 30.59% | 27.46% | 5.12 | <0.001 |
|  | *Fusarium* | 1.03% | 0.24% | 0.69% | 1.29% | 3.77 | <0.001 |
|  | *Sporobolomyces* | 1.14% | 0.70% | 0.39% | 0.38% | 3.61 | <0.001 |
|  | *Meyerozyma* | 1.96% | 0.28% | 0.48% | 1.34% | 3.87 | 0.001 |

Supplementary Table 4 Differences in taxonomic profiles of skin microbiomes between age groups during photoaging. The LDA score and *P values* are calculated by linear discriminant analysis effect size (LEfSe). The significance level is LDA score> 3, *P value* < 0.05.
