# Supplementary material for: New Insights Into the Skin Microbial Communities and Skin Aging
Source: Front Microbiol. 2020 Oct 26;11:565549. doi: 10.3389/fmicb.2020.565549 (PMC7649423; doi:10.3389/fmicb.2020.565549)
Supplement: Supplementary Table 5 — The selected genus-specific primer pairs in our study. [file Table_5.DOCX]

| Microbial genus | Sequence (5’–3’) | Reference |
| --- | --- | --- |
| *Corynebacterium* | Cory-F: GCTTGTTGGTGGGGTAATGG  Cory-R: TCACAAAACGCTTCGTCCCT | This study |
| *Staphylococcus* | Stap-F: GGCCGTGTTGAACGTGGTCAAATCA  Stap-R: TIACCATTTCAGTACCTTCTGGTAA | (Martineau et al., 2001) |
| *Lactobacillus* | Lac-F: AGCAGTAGGGAATCTTCCA  Lac-R: CACCGCTACACATGGAG | (Rinttilä et al., 2004) |
| *Cutibacterium* | Cuti-F: GAAGAACCTTACCTGGGTTTGAC  Cuti -R: CTGACGACAGCCATGCACC | This study |

Table S5 The selected genus-specific primer pairs in our study.
