# Supplementary material for: New Insights Into the Skin Microbial Communities and Skin Aging
Source: Front Microbiol. 2020 Oct 26;11:565549. doi: 10.3389/fmicb.2020.565549 (PMC7649423; doi:10.3389/fmicb.2020.565549)
Supplement: Supplementary Table 6 — Multitest correction for the statistics (P-value) for clinical skin parameters of cheeks. [file Table_6.DOCX]

| Skin Parameters | *P value* | FDR P | Bonferroni P |
| --- | --- | --- | --- |
| Spots | ＜0.001 | ＜0.001 | ＜0.001 |
| UV Spots | ＜0.001 | ＜0.001 | ＜0.001 |
| Brown Spots | ＜0.001 | ＜0.001 | ＜0.001 |
| Red Areas | ＜0.001 | ＜0.001 | ＜0.001 |
| Wrinkles | ＜0.001 | ＜0.001 | ＜0.001 |
| Texture | ＜0.001 | ＜0.001 | ＜0.001 |
| Pores | ＜0.001 | ＜0.001 | ＜0.001 |
| Porphyrins | ＜0.001 | ＜0.001 | ＜0.001 |

Table S6: Multitest correction for the statistics (P value) for clinical skin parameters of cheeks. The significance level was 0.05.
