# Supplementary material for: New Insights Into the Skin Microbial Communities and Skin Aging
Source: Front Microbiol. 2020 Oct 26;11:565549. doi: 10.3389/fmicb.2020.565549 (PMC7649423; doi:10.3389/fmicb.2020.565549)
Supplement: Supplementary Table 7 — Multitest correction for the statistics (P-value) for species richness indices (Chao 1 indices). [file Table_7.DOCX]

| Group | Children | Youth | Middle Age | Elderly | *P* value | FDR P | Bonferroni P | |
| --- | --- | --- | --- | --- | --- | --- | --- | --- |
| 16s IA | 856.42 | 846.22 | 965.92 | 1108.28 | ＜0.01 | ＜0.01 | ＜0.01 |  |
| 16s PA | 1002.40 | 1033.02 | 1115.73 | 1000.28 | 0.05 | 0.10 | 0.31 |  |
| ITs IA | 639.74 | 691.82 | 1063.00 | 833.72 | ＜0.01 | ＜0.01 | ＜0.01 |  |
| ITs PA | 912.12 | 928.35 | 849.76 | 1084.73 | 0.04 | 0.07 | 0.24 |  |

Table S7:Multitest correction for the statistics (P value) for species richness indices (Chao 1 indices). We demonstrated the median of the Chao 1 indices in different groups in the processes of intrinsic aging(IA) and photoaging(PA). The significance level was 0.05.
