# Supplementary material for: New Insights Into the Skin Microbial Communities and Skin Aging
Source: Front Microbiol. 2020 Oct 26;11:565549. doi: 10.3389/fmicb.2020.565549 (PMC7649423; doi:10.3389/fmicb.2020.565549)
Supplement: Supplementary Table 8 — Multitest correction for the statistics (P-value) for Spearman correlation analysis between skin photoaging related dominant genera. [file Table_8.DOCX]

| ID | *P* value | FDR P |
| --- | --- | --- |
| Streptococcus-Neisseria | <0.01 | <0.01 |
| Staphylococcus-Cutibacterium | <0.01 | <0.01 |
| Staphylococcus-Streptococcus | <0.01 | <0.01 |
| Cutibacterium-Streptococcus | <0.01 | <0.01 |
| Staphylococcus-Neisseria | <0.01 | <0.01 |
| Malassezia-Staphylococcus | <0.01 | <0.01 |
| Cutibacterium-Neisseria | <0.01 | <0.01 |
| Malassezia-Cutibacterium | <0.01 | <0.01 |
| Lactobacillus-Neisseria | <0.01 | <0.01 |
| Malassezia-Candida | <0.01 | <0.01 |
| Candida-Cutibacterium | <0.01 | <0.01 |
| Malassezia-Streptococcus | <0.01 | <0.01 |
| Staphylococcus-Lactobacillus | <0.01 | 0.01 |
| Candida-Staphylococcus | 0.01 | 0.01 |
| Candida-Neisseria | 0.02 | 0.02 |
| Lactobacillus-Cutibacterium | 0.02 | 0.03 |
| Lactobacillus-Streptococcus | 0.02 | 0.03 |
| Malassezia-Neisseria | 0.08 | 0.08 |
| Candida-Streptococcus | 0.11 | 0.11 |
| Malassezia-Lactobacillus | 0.12 | 0.12 |
| Candida-Lactobacillus | 0.46 | 0.46 |

Table S8: Multitest correction for the statistics (*P* value) for Spearman correlation analysis between skin photoaging related dominant genera. The significance level was 0.05.
