# Supplementary material for: New Insights Into the Skin Microbial Communities and Skin Aging
Source: Front Microbiol. 2020 Oct 26;11:565549. doi: 10.3389/fmicb.2020.565549 (PMC7649423; doi:10.3389/fmicb.2020.565549)
Supplement: Supplementary Table 9 — Multitest correction for the statistics (P-value) for Spearman correlation analysis between skin intrinsic aging related dominant genera. [file Table_9.DOCX]

| ID | *P* value | FDR P |
| --- | --- | --- |
| Neisseria-Streptococcus | <0.01 | <0.01 |
| Staphylococcus-Corynebacterium | <0.01 | <0.01 |
| Malassezia-Corynebacterium | <0.01 | <0.01 |
| Malassezia-Neisseria | <0.01 | <0.01 |
| Malassezia-Staphylococcus | <0.01 | 0.01 |
| Malassezia-Streptococcus | 0.01 | 0.01 |
| Neisseria-Corynebacterium | 0.01 | 0.02 |
| Corynebacterium-Streptococcus | 0.03 | 0.04 |
| Candida-Staphylococcus | 0.04 | 0.04 |
| Candida-Corynebacterium | 0.07 | 0.09 |
| Candida-Neisseria | 0.11 | 0.12 |
| Candida-Streptococcus | 0.14 | 0.15 |
| Candida-Malassezia | 0.15 | 0.16 |
| Staphylococcus-Neisseria | 0.24 | 0.25 |
| Staphylococcus-Streptococcus | 0.50 | 0.50 |

Table S9: Multitest correction for the statistics (*P* value) for Spearman correlation analysis between skin intrinsic aging related dominant genera. The significance level was 0.05.
